# Evaluation of serum levels of C-reactive protein, D-Dimer and Autologous Serum Skin Test in patients with Chronic Spontaneous Urticaria in a Brazilian tertiary center : a cross-sectional study^☆^^☆☆^

**DOI:** 10.1016/j.abd.2020.07.006

**Published:** 2021-02-01

**Authors:** Roberta Fachini Criado, Carolina Games Bensi, Paulo Ricardo Criado, Marina Teixeira Henriques, Beatriz Alessi Rodrigues de Espindola, Carlos D'Apparecida Machado Filho

**Affiliations:** a Department of Dermatology, Faculdade de Medicina do ABC, Santo André, SP, Brazil; b Infusion Center, Faculdade de Medicina do ABC, Santo André, SP, Brazil; c Faculdade de Medicina do ABC, Santo André, SP, Brazil; d Pediatric Allergy Department, Faculdade de Medicina do ABC, Santo André, SP, Brazil

**Keywords:** Angioedema, Pharmacological biomarkers, Urticaria

## Abstract

**Background:**

The pathophysiology of urticaria is still poorly understood. Recent studies demonstrate that the activation of coagulation is correlated with the clinical activity of Chronic Spontaneous Urticaria. Coagulation and inflammation are strongly linked.

**Objectives:**

To correlate the severity and activity of Chronic Spontaneous Urticaria with the levels of D-dimer, C-reactive protein, and autologous serum test in patients with Chronic Spontaneous Urticaria.

**Methods:**

The study included 55 patients diagnosed with chronic spontaneous urticaria. D-dimer levels were measured using enzyme-linked fluorescent assay and C-reactive protein levels were measured using the nephelometric method; autologous serum testing was performed on patients who discontinued antihistamine therapy. The severity of the disease was assessed using the urticaria activity score.

**Results:**

patients with severe, spontaneous, and difficult-to-control chronic urticaria had elevated serum levels of D-dimer, as well as a positive autologous serum test. Little correlation was demonstrated between the severity of chronic spontaneous urticaria and the levels of C-reactive protein.

**Conclusion:**

The authors concluded that patients with severe Chronic Spontaneous Urticaria showed signs of activated fibrinolysis. Most patients with high clinical scores had high D-dimer values. Patients with positive results for the autologous serum test also had more severe Chronic Spontaneous Urticaria and needed more drugs to control the disease. Finally, little correlation was found between C-reactive protein levels and disease severity.

**Study limitations:**

The main limitation was the small sample of patients. In the present patients, it was demonstrated that serum D-dimer levels and the autologous serum test can act as predictive markers of severity and activity of Chronic Spontaneous Urticaria.

## Introduction

It is estimated that the prevalence of chronic urticaria (CU) in the general population ranges from 0.5% to 5%. The incidence of CU was estimated at 1.4% per year. Some patients with this disease may experience urticaria and angioedema, occurring simultaneously or separately.^1^ Chronic Spontaneous Urticaria greatly impacts quality of life, due its to debilitating and unpredictable symptoms.^1^

In recent years, some authors have demonstrated the activation of the coagulation system in patients with CU due to thrombin generation, initiated by the increased expression of the coagulation tissue factor in eosinophils. This determines a possible contribution to the increase in capillary permeability. These patients generally have elevated serum coagulation and fibrinolysis markers, such as the prothrombin fragment_1+2_ and D-dimer, whose levels appear to be correlated with the severity of CU.^2^ In animal models, thrombin increases capillary permeability by direct action on the endothelium and also indirectly, inducing the release of pro-inflammatory mediators by mast cells, which leads to an increase in C5a in the absence of C3 without activation of the first part of the complement cascade.^2^, ^3^, ^4^ It is possible that autoantibodies and the coagulation cascade act in synergy in some patients with chronic urticaria.^2^

A systematic review conducted by Rabelo-Filardi et al. involving 34 published studies on spontaneous CU (CSU) concluded that the clinical severity of CU can predict the duration of the disease and that laboratory parameters, such as the elevation of serum levels of prothrombin fragments_1+2_, D-dimer, and C-reactive protein (CRP), can also convey the severity of the disease and its resistance to conventional treatment.^5^ Patients with more severe symptoms may have more persistent illnesses. Subsequently, other authors carried out more extensive reviews on various biomarkers of CSU.^6^, ^7^, ^8^

Coagulation and inflammation are strongly connected to each other. For example, inflammatory cytokines such as interleukin-6 (IL-6) and GM-CSF induce tissue factor (TF) expression in eosinophils, while the factor VII + FT activated complex binds to protease-activated receptor 2 (PAR-2) and regulates the inflammatory response. Therefore, it is feasible that C-reactive protein, a widely used inflammatory response marker, is associated with Chronic Spontaneous Urticaria activity similar to blood clotting markers.^4^

Studies involving the behavior of serum levels of CRP, D-dimer, and/or autologous serum skin test (ASST) results in Latin American CSU patients are rare in the indexed literature.^9^, ^10^

This study aimed to investigate the D-dimer and CRP plasma levels, as well as the positivity of the ASST and the clinical severity of CSU in this group of patients, and to determine which of these complementary tests may be relevant to the clinical severity of this disease in Brazilian patients treated at a tertiary center.

## Methods

This was a cross-sectional study, in which 55 patients with a diagnosis of Chronic Spontaneous Urticaria were followed-up at the urticaria outpatient clinic in the dermatology department of the Faculdade de Medicina do ABC.

Inclusion criteria: patients followed-up on an outpatient basis with a clinical diagnosis of CSU and recurrence of hives at least four times a week at the time of inclusion in the study. After obtaining the consent form, the standard form applied to all patients followed-up at the clinic was completed. CRP and D-dimer were measured in all patients in the first consultation, whereas the ASST was performed in most of them in the following consultation, since they had to undergo a period without the use of antihistamines, as previously recommended by Konstantinou et al.,^11^ hindering the execution of this test in some patients. All ASST were performed in the morning on the patient's second visit to the service. Patients were followed-up in monthly consultations.

Study participants were instructed to complete the Urticaria Activity Score (UAS) at home. The UAS values on the day before the ASST were relevant for comparing CSU severity with the results of these tests. Disease activity was assessed using the EAACI/GA2LEN/EDF1 activity score, which consists of papules score (none = 0, mild [<20 lesions in 24 hours] = 1, moderate [20–50 lesions in 24 hours] = 2, and intense [>50 lesions in 24 hours] = 3) and pruritus score (no pruritus = 0, mild [present, but not irritating or problematic] = 1, moderate [problematic, but does not interfere in activities of daily living or sleep] = 2, and intense [severe itching, interferes with normal daily activity or sleep] = 3). The disease activity was scored from 0 to 6, according to the previous consensus published by Zurberbier et al., at the time the study was conducted.^12^ Taking this as a basis, disease activity was classified as follows: UAS 0 was considered controlled urticaria; UAS 1 to 3, low; and UAS 4 to 6, high.

The exclusion criteria were the presence of any disease or medication used that altered the coagulation cascade (oral or parenteral anticoagulants, antifibrinolytics, antiplatelet agents), uncertain diagnosis of CSU (due to the presence of signs or symptoms such as fever, arthralgia, acute lymphadenopathy, chronic infectious diseases, pregnancy, or skin signs of urticarial vasculitis during physical examination), and current or past history of thrombosis.

CRP serum concentration protein was measured by the nephelometric method (Dade Behring Inc. – Newark, DE, United States) in the central laboratory of this institution. Elevated serum CRP was defined as ≥ 5.0 mg/L.

D-dimer levels were measured in patients using enzyme-bound fluorescent assay (ELFA) (BioMérieux equipment, model VIDAS®) and the results presented in mg/L FEU (fibrinogen equivalent unit). Values ≤ 0.50 mg/L FEU were considered negative and >0.50 mg/L FEU, as positive.

All patients in the study were given first-line treatment for the disease, based on second-generation antihistamines (anti-H1) in single to quadruplicate doses. For patients classified as UAS > 1 and not responsive to treatment with daily quadruplicate antihistamine after a minimum duration of one month, an adjuvant therapy with one of the following third-line drugs was initiated: cyclosporine, dapsone, colchicine, hydroxychloroquine, montelukast, and oral prednisone for seven days in hives and/or angioedema flares.

To quantify the response of each patient to the established treatment, a response score was created at the time of remission of the disease (UAS = 0, for at least 30 days): grade 1, when the patient presented remission with only one dose of antihistamine medication; grade 2, if the patient obtained urticaria control with two doses of anti-H1; grade 3, if urticaria was controlled with three doses of anti-H1; grade 4, if urticaria was controlled with four doses of anti-H1; and grade 5, if control of this disease occurred with four doses of anti-H1 combined with an adjuvant medication.

Patients with difficulty in controlling CSU and needing high doses of medication for this were considered to have a low response score, whereas those with remission using few doses of medication were classified as having a high response score.

Qualitative variables such as gender, classification of CSU severity (low/high), adjuvant medication (yes/no), and ASST positivity were represented by absolute (n) and relative (%) frequencies. Normally distributed quantitative continuous variables, such as CRP and D-dimer, were calculated using mean values ± standard deviation (SD), median, and minimum and maximum values. Student's *t*-test was used to compare the mean values of UAS in relation to patients with positive *vs*. negative ASST, for the mean values of D-dimer among patients who needed adjuvant medication or not, for the mean values of D-dimer among patients with a high *vs*. low clinical score, and for mean D-dimer values in patients with CSU with angioedema *vs*. CSU without angioedema. Pearson's correlation coefficient was used to provide a measure of the strength of linear associations between the variables D-dimer and UAS, D-dimer and number of drugs needed to control CSU, CRP *vs*. UAS, CRP *vs*. clinical score (number of doses), and CRP *vs*. number of antihistamines.

The association between patients with positive and negative ASST *vs*. high and low UAS was assessed using Fisher's exact test. The odds ratios were calculated. SPSS v. 17, Minitab 16, and Excel Office 2010 software were used for the statistical analysis. The level of significance considered in the comparative analyses was 5% (p < 0.05). The project was approved by the ethics committee under opinion No. 2853158.

## Results

### Demographic data

Of the total of 55 patients with CSU, 40 (72.72%) were female and 15 (27.27%) were male. Regarding the age of the patients (18 to 72 years), the mean age at the initial consultation was 41 ± 14.02 years (mean ± SD). Of the 55 patients, 26 (47.22%) had urticaria associated with angioedema.

### Disease activity (severity)

The UAS, adapted from Zuberbier et al.,^1^ was used to assess CSU severity. In order to find a relationship between laboratory parameters and urticaria severity, the variables ASST, D-dimer, and CRP were related to the UAS of the day before the exams. To facilitate the analysis, patients were stratified into groups: low score (UAS 1 to 3) and high score (UAS 4 to 6). Twenty-five patients were classified as low score (45%) and 30 patients as high score (55%; Table 1).

**Table 1:** Distribution of patients according to the UAS obtained the day before the second visit.

UAS Group	Number	(%)
Low	25	45%
High	30	55%

### CSU severity vs. ASST

Of the 55 patients, it was possible to perform ASST in 29. The others were unable to suspend antihistamine therapy for the period determined by the method (Konstantinou et al.).^11^ The results of the mean UAS values for patients with positive ASST (4.79) were significantly higher when compared with the mean UAS values of patients with negative ASST results (3.3) (Student's *t*-test; p < 0.05).

The results in Table 2 indicate that there was a significant association between ASST and UAS (Fisher's exact test; p = 0.017). A total of 61% of patients with positive ASST met the inclusion criteria in the group of high scores for CSU severity, while 73% of patients with negative ASST were in the group of low scores. The odds ratio between the variables indicated a result equal to 4.30, *i.e*., the odds of a positive ASST patient having a high score for urticaria activity was 4.3 times greater when compared with a negative ASST patient.

**Table 2:** Results of the autologous serum skin test (ASST) and Urticaria Activity Score (UAS) in 29 patients who underwent the ASST.

Group	Positive ASST	Negative ASST
High	61%	39%, p < 0.001
Low	27%	73%

### D-dimer vs. CSU severity

**Figure 1:**
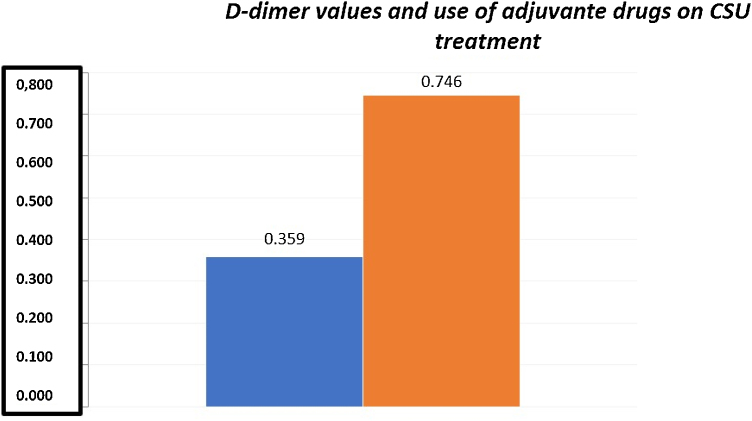
Patients who required the use of adjuvant medication according to D-dimer levels, blue bar - no need of adjuvant medication.

The mean D-dimer values in the present sample were 0.85 ± 0.324 mg/L FEU (mean ± SD). Among the 55 patients with CSU, 22 presented high plasma levels of this variable (values between 0.55–1.48 mg/L FEU). The mean D-dimer for the group of patients who did not need adjuvant medication (0.359 ± 0.206 mg/L FEU) was significantly lower than the mean D-dimer for the group of patients who needed an adjuvant medication (0.746 ± 0.444 mg/L FEU) for urticaria control (Student's *t*-test; p < 0.001; Fig. 1).

**Figure 2:**
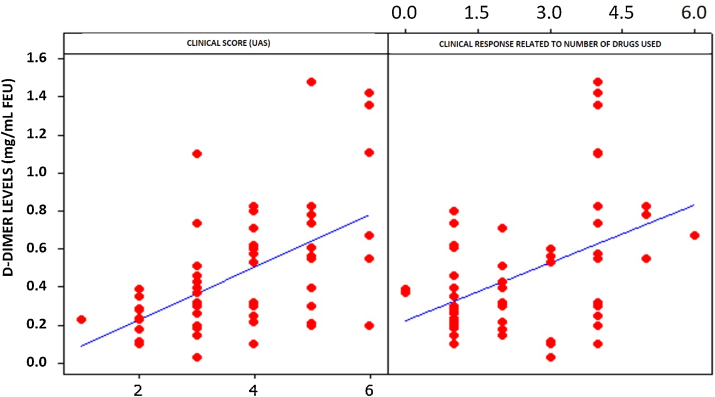
Correlation between D-dimer levels, number of medications used, and Urticaria Activity Score (UAS).

Statistical analysis of D-dimer and UAS levels demonstrated a strong and positive relationship between these two variables. A 53.1% correlation was observed (Fig. 2). A significant correlation was also observed between the number of drugs needed to control CSU and elevated plasma levels of D-dimer (Fig. 2). Both correlations were significant (p < 0.01).

A significantly lower mean D-dimer value was observed in patients with a low clinical score (0.321 ± 0.221 mg/L FEU) when compared with patients with a high clinical score (0.598 ± 0.374 mg/L FEU; Student's *t*-test; p = 0.002).

Elevated serum D-dimer levels (>0.5 mg/mL FEU) were observed in 22 patients. Among them, 18 (81.81%) were classified as having an CSU with a high activity score and poor disease control (UAS 4 to 6). Twelve patients (54.5%) had a high UAS score (4 to 6), and 32.7% of these patients needed three or more drugs in combination to achieve CSU remission during follow-up.

When assessing patients with CSU and angioedema or CSU without angioedema, no statistical significance was found in relation to plasma levels of D-dimer in both groups (Student's *t*-test; p = 0.061). The mean values and standard deviations of D-dimer were, respectively, 0.563 ± 0.369 mg/mL FEU and 0.390 ± 0.297 mg/mL FEU. While the difference was not significant, a trend towards a higher D-dimer result for patients with CSU and angioedema was observed.

### CRP vs. UAS

**Figure 3:**
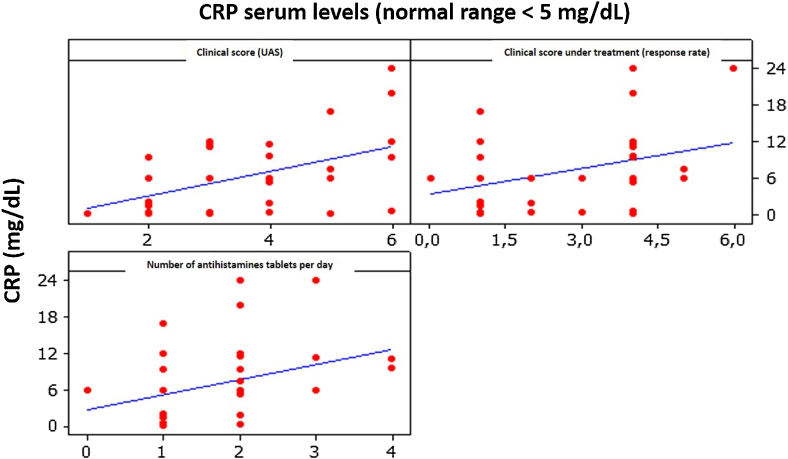
Correlation between CRP, clinical score, and medications used.

The correlation between CRP and UAS was moderate and positive, being 0.496 (p < 0.01), indicating that an increase in CRP levels is accompanied by a slight increase in UAS (Fig. 3).

## Discussion

In the last decade, several studies have been carried out to evaluate the role of coagulation activation in the pathophysiology of CSU. Plasma levels of D-dimer, a marker for the formation of fibrin and its subsequent degradation, have been proposed as a biomarker of activity and severity in CSU. This issue has been widely reviewed by Kolkhir et al.,^6^ Puxeddu et al.,^7^ and Deza et al.,^8^ and these authors concluded that CRP and D-dimer may be related to the severity of the CSU in some patients. However, the literature review is composed of different studies that applied different methods (case series, non-blinded studies, case reports), making it difficult to reach a final conclusion on this issue, especially with regard to the real usefulness of these complementary exams to predict the severity of the CSU in different patient cohorts.

In 2007, Asero et al.^13^ published one of the first studies that evaluated 37 patients with chronic urticaria with a measurement of prothrombin fragments_1+2_, D-dimer, factor XIIa, and factor VIIa. In that study, the authors found high mean D-dimer levels in patients with CU, as opposed to the healthy control group, also demonstrating markers of fibrinolysis activation in their patients.^13^ It was then proven by the authors that the activation of coagulation and, consequently, the presence of D-dimer, can be correlated with disease severity.^13^ Similar findings related to plasma D-dimer levels and CSU severity were observed in the present study.

An increase in serum levels of CRP is believed to occur, probably due to the cutaneous expression of IL-6, in patients with severe CSU.^2^ The correlation between serum CRP levels and coagulation markers suggests an interrelation between inflammation and coagulation in the pathogenesis of chronic urticaria. However, the exact role of this phenomenon (activation of coagulation and fibrinolysis) acting as the centerpiece of the pathophysiology of the disease or epiphenomenon acting as an inflammation amplifier is still unclear. In the present study, no statistically significant increase in CRP was observed.

In an immunohistochemical study, Cugno et al.^2^ reported that eosinophils in biopsies expressed TF in patients with CU, which were activated probably by IL-6 and GM-CSF in these cells, and that TF was involved as initiator of the extrinsic pathway of the coagulation cascade, in the dermis milia.^2^

These authors suggested that D-dimer is a superior biomarker in terms of sensitivity to fibrin degradation product (FDP) and to CRP, in addition to demonstrating that FDP, D-dimer, and CRP levels can be significantly correlated to disease activity in some patients with CU.^2^ Nonetheless, in the present study, the correlation between serum CRP levels and the severity of CSU was inconsistent. This fact can be justified by some limitations in the present study: less ethnically diverse population when compared with Asia and Europe; the small number of patients included in this analysis, as this is a specialized urticaria center; and the short period of analysis (three years).

With regard to ethnic aspects, in Brazil there is frequent racial mixing, in contrast to other countries in Latin America. This fact can influence the balance of genders in patients with CSU – the present study found a frequency of 72.72% female patients, moderately more prevalent in relation to patients seen in Buenos Aires, which is another large urban area in Latin America, Parisi et al. observed that 66.7% of patients with CU were female in an observational study conducted in the same decade.^14^

Kirchhof et al.^15^ published two case reports, one on cutaneous polyarteritis nodosa (cutaneous arteritis) and the other on atypical recurrent urticaria, where the same variables as the present study were evaluated: CRP and D-dimer values during disease activity. Interestingly, serum C-reactive protein levels did not correlate with disease activity and the authors found levels within normal ranges during clinically active disease.^14^ In these two reported cases, CRP levels were normal and D-dimer levels were high, indicating that CRP, a marker of acute inflammation, may be less sensitive than levels of D-dimer, a generator of fibrinolytic activity. This fact was also observed in patients with CSU in the present study. CSU remission may be related to antihistamine treatment and/or adjuvant medications; however, spontaneous remissions occur in 30%–50% of patients within one year of disease progression and another 20% within five years.^16^ Approximately 20% of patients with CSU maintain disease activity even after five years of evolution.^16^ Several studies have shown that patients with more severe symptoms may have a more persistent course of the disease. Almost half of patients with chronic spontaneous urticaria lasting over six months are likely to still have clinical manifestations ten years later.^16^ In the present study, a relationship was observed between the severity of the CSU, plasma levels of D-dimer, UAS, and the number of antihistamines and adjuvant drugs necessary to obtain clinical control of the CSU.

In a large retrospective observational study in Barcelona (Spain), Curto-Barredo et al.^17^ studied 549 patients with CU and found that more than 75% of them were refractory to first-line treatment with licensed doses of anti-H1. These authors demonstrated that baseline UAS was the only parameter capable of predicting refractoriness to anti-H1. The present findings were in agreement with those by Curto-Barredo et al.,^17^ and the authors observed that patients with a high CSU score needed more drugs to control the disease.

### Limitations

The sample was small in relation to the prevalence of the disease. Larger studies are needed to clarify the importance of using biomarkers in CSU.

## Conclusions

This study demonstrated that patients with positive ASST had a high urticaria activity score, showing that patients with autoimmune/autoreactive urticaria have a more severe and more difficult to treat CSU.

A strong and directly proportional relationship was observed between plasma D-dimer levels and the clinical activity score, although this same relationship was not observed between serum CRP levels and the clinical score, indicating that D-dimer is a more reliable variable than CRP as a marker of severity in chronic urticaria.

## Financial support

None declared.

## Authors’ contributions

Roberta Fachini Criado: Statistical analysis; approval of the final version of the manuscript; conception and planning of the study; elaboration and writing of the manuscript; obtaining, analyzing, and interpreting the data; effective participation in research orientation; intellectual participation in propaedeutic and/or therapeutic conduct of studied cases; critical review of the literature; critical review of the manuscript.

Carolina Games Bensi: Design and planning of the study, drafting and editing of the manuscript, collection, analysis, and interpretation of data, intellectual participation in propaedeutic and/or therapeutic conduct of studied cases, critical review of the literature.

Paulo Ricardo Criado: Statistical analysis; approval of the final version of the manuscript; conception and planning of the study; elaboration and writing of the manuscript; obtaining, analyzing, and interpreting the data; effective participation in research orientation; intellectual participation in propaedeutic and/or therapeutic conduct of studied cases; critical review of the literature; critical review of the manuscript.

Marina Teixeira Henriques: Design and planning of the study, drafting and editing of the manuscript, collection, analysis, and interpretation of data, critical review of the literature.

Beatriz Alessi Rodrigues de Espindola: Statistical analysis, approval of the final version of the manuscript, design and planning of the study, drafting and editing of the manuscript, collection, analysis, and interpretation of data, intellectual participation in propaedeutic and/or therapeutic conduct of studied cases.

Carlos D'apparecida Machado Filho: Effective participation in research orientation, critical review of the manuscript.

## Conflicts of interest

None declared.
